# Identification of candidate genes associated with fibromyalgia susceptibility in southern Spanish women: the al-Ándalus project

**DOI:** 10.1186/s12967-018-1416-8

**Published:** 2018-02-27

**Authors:** Fernando Estévez-López, Daniel Camiletti-Moirón, Virginia A. Aparicio, Víctor Segura-Jiménez, Inmaculada C. Álvarez-Gallardo, Alberto Soriano-Maldonado, Milkana Borges-Cosic, Pedro Acosta-Manzano, Rinie Geenen, Manuel Delgado-Fernández, Luis J. Martínez-González, Jonatan R. Ruiz, María J. Álvarez-Cubero

**Affiliations:** 1 Department of Physical Education and Sport, Faculty of Sport Sciences, University of Granada, Carretera de Alfacar, s/n, 18011 Granada, Spain; 2 Department of Psychology, Faculty of Social and Behavioural Sciences, Utrecht University, Utrecht, The Netherlands; 3 Department of Physical Education, Faculty of Education Sciences, University of Cádiz, Cádiz, Spain; 4 Department of Physiology, Faculty of Pharmacy, Faculty of Sport Sciences and Institute of Nutrition and Food Technology, University of Granada, Granada, Spain; 5 Department of Education, Faculty of Education Sciences, University of Almería, Almería, Spain; 6 SPORT Research Group (CTS-1024), CERNEP Research Center, University of Almería, Almería, Spain; 7 Liquid Biopsy and Metastasis Research Group and Genomic Unit, GENyO (Pfizer-University of Granada, Andalusian Government Centre for Genomics and Oncological Research), Granada, Spain; 8 PROFITH “PROmoting FITness and Health Through Physical Activity” Research Group, Department of Physical Education and Sport, Faculty of Sport Sciences, University of Granada, Granada, Spain; 9 Department of Biochemistry and Molecular Biology III, Faculty of Medicine, University of Granada, Granada, Spain

**Keywords:** Alleles, Caucasian, Chronic pain, COMT gene, Epidemiology, GCH1 gene, OPRM1 gene

## Abstract

**Background:**

Candidate-gene studies on fibromyalgia susceptibility often include a small number of single nucleotide polymorphisms (SNPs), which is a limitation. Moreover, there is a paucity of evidence in Europe. Therefore, we compared genotype frequencies of candidate SNPs in a well-characterised sample of Spanish women with fibromyalgia and healthy non-fibromyalgia women.

**Methods:**

A total of 314 women with a diagnosis of fibromyalgia (cases) and 112 non-fibromyalgia healthy (controls) women participated in this candidate-gene study. Buccal swabs were collected for DNA extraction. Using TaqMan™ OpenArray™, we analysed 61 SNPs of 33 genes related to fibromyalgia susceptibility, symptoms, or potential mechanisms.

**Results:**

We observed that the rs841 and rs1799971 GG genotype was more frequently observed in fibromyalgia than in controls (*p* = 0.04 and *p* = 0.02, respectively). The rs2097903 AT/TT genotypes were also more often present in the fibromyalgia participants than in their control peers (*p* = 0.04). There were no differences for the remaining SNPs.

**Conclusions:**

We identified, for the first time, associations of the rs841 (guanosine triphosphate cyclohydrolase 1 gene) and rs2097903 (catechol-*O*-methyltransferase gene) SNPs with higher risk of fibromyalgia susceptibility. We also confirmed that the rs1799971 SNP (opioid receptor μ1 gene) might confer genetic risk of fibromyalgia. We did not adjust for multiple comparisons, which would be too stringent and yield to non-significant differences in the genotype frequencies between cases and controls. Our findings may be biologically meaningful and informative, and should be further investigated in other populations. Of particular interest is to replicate the present study in a larger independent sample to confirm or refute our findings. On the other hand, by including 61 SNPs of 33 candidate-genes with a strong rationale (they were previously investigated in relation to fibromyalgia susceptibility, symptoms or potential mechanisms), the present research is the most comprehensive candidate-gene study on fibromyalgia susceptibility to date.

**Electronic supplementary material:**

The online version of this article (10.1186/s12967-018-1416-8) contains supplementary material, which is available to authorized users.

## Background

Fibromyalgia is a disease in which people experience chronic diffuse musculoskeletal pain that is usually accompanied by other symptoms such as fatigue, unrefreshed sleep, and cognitive problems [1–4]. This disease is nine times more common in women than in men [5]. Family aggregation suggests but not confirm genetic susceptibility to fibromyalgia [6]. Variations in neurotransmitter-related genes increase susceptibility to fibromyalgia via hypersensitivity to peripheral painful stimulus by the central nervous system (CNS) [7]. Catechol-*O*-methyltransferase (COMT), the most widely studied gene in fibromyalgia, is involved in degrading catecholamines and several other neurotransmitters and, therefore, in modulating pain perception by the CNS. The association between COMT single nucleotide polymorphisms (SNPs) and fibromyalgia susceptibility is controversial [7, 8].

Guanosine triphosphate cyclohydrolase 1 (GCH1) and opioid receptor μ1 (OPRM1) are candidate neurotransmitter-related genes that may confer fibromyalgia susceptibility [9, 10]. GCH1 gene participates in the synthesis of dopamine and serotonin. In Korean individuals, the rs841 SNP was associated with discomfort with a tender point examination [9]. OPRM1 gene encodes μ-opioid receptor that bids opiates. In the Turkish population, a study identified that rs1799971 SNP is associated with fibromyalgia susceptibility [10]. However, whether GCH1 and OPRM1 genes are associated with fibromyalgia susceptibility in the Caucasian population is unknown.

Candidate-gene studies on fibromyalgia susceptibility often include a small number of SNPs (e.g., [9–11]), which is a limitation [12, 13]. Additionally, there is a paucity of evidence in Europe. The present candidate-gene study compared genotype frequencies of candidate SNPs in a well-characterised sample of Spanish women with fibromyalgia (cases) vs. healthy non-fibromyalgia women (controls). We, therefore, analysed SNPs that had been previously investigated in relation to fibromyalgia susceptibility, symptoms, or potential mechanisms.

## Methods

### Participants

The participants were recruited mainly via fibromyalgia associations from Andalusia (southern Spain). The fibromyalgia patients invited to non-fibromyalgia acquaintances with similar sociodemographic characteristics to participate in the study as controls. All participants signed an informed consent form. The Ethics Committee of the *Virgen de las Nieves* Hospital (Granada, Spain) approved the study. We followed the ethical guidelines of the Declaration of Helsinki.

The fibromyalgia participants had been previously diagnosed with fibromyalgia by a professional rheumatologist and met the 1990 American College of Rheumatology (ACR) criteria for fibromyalgia, which was further confirmed by a tender point examination [1]. Controls neither had a medical diagnosis of fibromyalgia nor fulfilled the 1990 ACR criteria.

### Genetic analysis

The participants were genotyped for 61 SNPs (online Additional file 1: Table S1) that had been previously investigated in relation to fibromyalgia susceptibility, symptoms, or potential mechanisms (online Additional file 1: Table S2). As described elsewhere [14, 15], we collected buccal mucosa cells and we performed DNA non-organic extraction (proteinase K and salting-out) procedures. Afterwards, we conducted a spectrophotometric quantification (NanoDrop 2000c, ThermoFisher). All the samples were standardised to 50 ng/μL and they had an A260/230 ratio between 1.7 and 1.9. The samples in low concentration values were processed by Genomiphi™ V2 DNA Amplification Kit (Sigma Aldrich). Until being processed, all the samples were stored at − 20 °C. The OpenArray^®^ genotyping was performed according to the manufacturer’s protocol using Accufill™ system liquid robot that adds 1.2 μl of genomic DNA sample and 3.8 µl of the following reagents: 2× TaqMan^®^ Universal PCR Master Mix, No AmpErase^®^ UNG, 20× Primer and TaqMan^®^ Probe (FAM™ dye) mix, and sterile-filtered water. The online Additional file 1: Table S3 shows the manufacturer thermal cycling conditions.

Plates include a NTC for each SNP in the analysis, and each plate has a total of 48 samples. Online Additional file 1: Tables S1 and S4 provide further details about the TaqMan^®^ OpenArray^®^ custom assay designs and the 61 analysed SNPs, respectively. We performed a TaqMan™ OpenArray™ Genotyping Plate, Custom Format 64 QuantStudio™ 12 K Flex. The data were analysed using the TaqMan^®^ Genotyper Software and downstream analysis using the AutoCaller™ Software.

### Statistical analysis

The Hardy-Weinberg equilibrium (HWE) and linkage disequilibrium (LD = *r*^*2*^ > 0.5) were checked for all the SNPs using SNPStats v3.0.1 [16]. Using SPSS for Mac v.20.0 (IBM, Armonk, NY, USA), we computed Pearson’s χ^2^ and logistic regression to analyse the differences between fibromyalgia and controls on SNPs genotype frequency. Significance was set at *p* < 0.05.

## Results

Online Additional file 1: Table S5 shows the characteristics of the participants included in the present study. Those SNPs that did not fulfil genotyping quality controls were excluded. Through this strict filtering out rule we excluded the following nine SNPs because they did not meet the HWE criterion: the rs6323, rs7911, rs136078, rs806377, rs1050450, rs3746544, rs4411417, rs7124442, and rs12620053. HWE and LD were confirmed for the remaining 52 SNPs, which were, therefore, included in the present study.

The rs841and rs1799971 GG genotypes were more frequently observed in fibromyalgia than in controls [odds ratio (OR) (95% confidence interval, 95% CI) = 1.66 (1.03–2.67), *p* = 0.04 and 1.72 (1.11–2.67), *p* = 0.02, respectively; Table 1]. The rs2097903 AT/TT genotypes were also present in the fibromyalgia participants more often than in their non-fibromyalgia peers [OR (95% CI) = 1.66 (1.02–2.71), *p* = 0.04; Table 1). There were no differences for the remaining 49 SNPs (online Additional file 1: Table S6).

**Table 1:** Genotype frequencies of single nucleotide polymorphisms (SNP) in fibromyalgia (FM) and non-fibromyalgia (control) women

SNP (gene)	Genotype/allele	FM, *n* (%)	Control, *n* (%)	OR (95% CI, lower to upper)	*p* value
rs841 (GCH1)	AA/AG	72 (23.2)	37 (33.3)	1.66 (1.03–2.67)	0.04
	GG	239 (76.8)	74 (66.7)		
	A	76 (12.2)	40 (18.0)	1.58 (1.04–2.40)	0.03
	G	546 (87.8)	182 (82.0)		
rs1799971 (OPRM1)	AA/AG	137 (44.2)	64 (57.7)	1.72 (1.11–2.67)	0.02
	GG	173 (55.8)	47 (42.3)		
	A	163 (26.3)	78 (35.1)	1.52 (1.09–2.11)	0.01
	G	457 (73.7)	144 (64.9)		
rs2097903 (COMT)	AA	64 (21.2)	34 (30.9)	1.66 (1.02–2.71)	0.04
	AT/TT	238 (78.8)	76 (69.1)		
	A	274 (45.4)	116 (52.7)	1.34 (0.99–1.83)	0.06
	T	330 (54.6)	104 (47.3)		

Logistic regression analyses were conducted to calculate de odds ratio (OR) and 95% confidence interval (95% CI)

*COMT* catechol-*O*-methyltransferase gene, *GCH1* GTP cyclohydrolase 1 gene, *OPRM1* opioid receptor μ1 gene, *A* Adenine, *C* Cytosine, *G* Guanine, *T* Thymine

## Discussion

The present candidate-gene study (including 61 SNPs of 33 genes) is the largest conducted on fibromyalgia susceptibility. We identified, for the first time, that the rs841 (GCH1 gene) and rs2097903 (COMT gene) SNPs were associated with having fibromyalgia. We also confirmed that the rs1799971 SNP (OPRM1 gene) seems to confer genetic risk of fibromyalgia. The COMT gene is involved in neurotransmitters degradation while the GCH1 gene is related to their synthesis. The μ-opioid receptor, which is encoded by the OPRM1 gene, bids both endogenous and exogenous opiates. In line with previous literature [7], our findings suggest that an augmented processing of pain may be involved in the genetic susceptibility to fibromyalgia.

Vargas-Alarcón et al. [8] showed that genotype frequencies of the rs4680, rs4818, and rs6269 SNPs, but not rs2097903, were different between fibromyalgia and control participants. In contrast, we found that the rs2097903 SNP was different between the study cohorts; 78.8% of the patients, and only 69.1% of the controls were TT genotype carriers. Vargas-Alarcón et al. [8] excluded fibromyalgia participants with rheumatic comorbidities and control participants with chronic pain. Of note is that we corroborated the rheumatologist diagnosis of our fibromyalgia participants by fulfilling the 1990 ACR criteria and discarding it in controls. We included fibromyalgia participants with other rheumatic conditions and controls that experienced chronic pain if they did not fulfil the fibromyalgia diagnosis. Therefore, the ecological validity of our findings is high. Moreover, a later genome-wide association study [7] in the Spanish population also failed to replicate the findings by Vargas-Alarcón et al. [8].

GCH enzyme participates in nitric oxide (NO) production. It is widely recognised that NO plays a key role in health and disease by a substantial number of paths [17]. Among other roles, increased NO concentrations often lead to dorsal horn hyperexcitability [18]. Overall, our results are consistent with a previous study in a Korean population in which genotype frequencies of rs841, rs752688, rs4411417, and rs3783641 SNPs in the GCH1 gene were not associated with fibromyalgia susceptibility [9]. However, we found a significant association of the rs841 SNP and fibromyalgia susceptibility with 76.8% of the cases carrying the GG genotype vs. 66.7% of the controls. Interestingly, rs841 was the only SNP correlated to discomfort with a tender point examination in Korean patients [9]. Currently, no study in Caucasian population is available; therefore, future research testing associations of GCH1 gene SNPs, particularly the rs841, with fibromyalgia susceptibility in Caucasians is welcome.

A hypersensitive CNS also appears to have behavioural implications. An active lifestyle is associated with better health status in fibromyalgia [19–21]. However, impaired functioning of the anterior cingulate cortex and amygdala, among other neural areas, may yield to processing harmless movements as painful [22]. The activity of these structures is modulated by μ-opioid receptor availability, which is reduced in fibromyalgia [23]. In agreement with a study in Turkey [10], our findings suggest that the rs1799971 SNP confers fibromyalgia susceptibility, as most of the present patients’ sample were GG genotype (55.8%) and most of the controls were AA/AG (57.7%).

Most of the non-significant findings emerged in SNPs of sodium voltage-gated channel alpha subunit 9 (SCN9A) and adrenoceptor alpha 1A (ADRA1A) genes. Genotype frequencies of a SCN9A SNP (rs6754031) were, however, significantly different between fibromyalgia and controls in Mexico [24]. ADRA1A gene activates mitogenic responses and regulates growth and proliferation of many cells. In two subsamples (from Spain and Mexico), a previous study analysed associations of 4 ADRA1A SNPs [25], showing that only rs1383914 was associated with an increased risk of fibromyalgia susceptibility in the Spanish sample [25].

We did not include a replication sample; therefore, our findings must be interpreted with caution until they are corroborated or refuted in future studies conducted in independent samples. In order to prevent flares on pain, we collected mucosa cells using buccal swabs (i.e., a both low level of invasiveness and pain method), which on the other hand, precludes conducting a functional study (e.g., gene or protein expression). The unadjusted analyses for multiple comparisons are another limitation, which would yield to non-significant differences in the genotype frequencies of the SNPs. However, to conclude that there is no difference from a statistical point of view would be too stringent. Our findings may be biologically meaningful and informative, and should be further investigated in other populations. Additionally, the unbalanced size of the study cases and controls limits our statistical power. We included, however, more people in the smaller group (cases, *n* = 112) than most of previous studies [26].

## Conclusions

In conclusion, we identified associations of the rs841 (GCH1 gene) and rs2097903 (COMT gene) SNPs with higher risk of fibromyalgia. We also confirmed that rs1799971 SNP (OPRM1 gene) seem to confer higher susceptibility to fibromyalgia. Further studies are needed to confirm or refute the present findings.

## Additional file

**Additional file 1: Table S1.** TaqMan™ OpenArray™ custom assay designs of candidate gene SNPs included in the present study. **Table S2.** Rationale for the inclusion of the 64 single nucleotide polymorphisms (SNPs) in the present study. **Table S3.** Thermal cycling conditions. **Table S4.** Further details of the single nucleotide polymorphisms (SNP) included in the present study. **Table S5.** Socio-demographic and clinical characteristics of the study samples. **Table S6.** Genotype frequencies of single nucleotide polymorphisms (SNP) in fibromyalgia (FM) and non-fibromyalgia (controls, HC) women.

## Abbreviations

ACR: American College of Rheumatology
ADRA1A: adrenoceptor alpha 1A
CNS: central nervous system
COMT: catechol-*O*-methyltransferase
GCH1: guanosine triphosphate cyclohydrolase 1
HWE: Hardy–Weinberg equilibrium
NO: nitric oxide
OPRM1: opioid receptor μ1
SCN9A: sodium voltage-gated channel alpha subunit 9
SNPs: single nucleotide polymorphisms
