# Supplementary material for: Identification of candidate genes associated with fibromyalgia susceptibility in southern Spanish women: the al-Ándalus project
Source: J Transl Med. 2018 Feb 27;16:43. doi: 10.1186/s12967-018-1416-8 (PMC5828244; doi:10.1186/s12967-018-1416-8)
Supplement: Supplementary file 1 — Additional file 1: Table S1. TaqMan™ OpenArray™ custom assay designs of candidate gene SNPs included in the present study. Table S2. Rationale for the inclusion of the 64 single nucleotide polymorphisms (SNPs) in the present study. Table S3. Thermal cycling conditions. Table S4. Further details of the single nucleotide polymorphisms (SNP) included in the present study. Table S5. Socio-demographic and clinical characteristics of the study samples. Table S6. Genotype frequencies of single nucleotide polymorphisms (SNP) in fibromyalgia (FM) and non-fibromyalgia (controls, HC) women. [file 12967_2018_1416_MOESM1_ESM.docx]

Table S1. TaqMan™ OpenArray™ custom assay designs of candidate gene SNPs included in the present study

| **Gene** | **SNP** | **Custom assay** |
| --- | --- | --- |
| **ADRA1A** | rs574584 | C___2315104_10 |
|  | rs1048101 | C___2696454_30 |
|  | rs1383914 | C___2696575_1_ |
| **ADRB2** | rs1042713 | C___2084764_20 |
|  | rs1042714 | C___2084765_20 |
| **ADRB3** | rs4994 | C___2215549_20 |
| **APOE** | rs429358 | C___3084793_20 |
| **BDNF-AS** | rs6265 | C__11592758_10 |
|  | rs7124442 | C__27833027_10 |
| **CNR1** | rs806377 | Self designed |
| **COMT** | rs4633 | C___2538747_20 |
|  | rs6269 | C___2538746_1_ |
|  | rs165599 | C___2255335_10 |
|  | rs2097903 | C__16114953_10 |
|  | rs2254137 | C__11514151_20 |
|  | rs242940 | C___2544836_10 |
| **CREB1** | rs7209436 | C___1570087_10 |
| **CRHR1** | rs6280 | C____949770_10 |
|  | rs1800443 | C___7470708_20 |
| **DRD3** | rs1800955 | C___7470700_30 |
| **DRD4** | rs1360780 | C___8852038_10 |
|  | rs3800373 | C__27489960_10 |
| **FKBP5** | rs9296158 | C___1256775_10 |
|  | rs9470080 | C_____92160_10 |
|  | rs4906902 | C__11300465_10 |
|  | rs7911 | Self designed |
| **GABRB3** | rs841 | C___9866639_10 |
| **GBP1** | rs752688 | C___9866644_10 |
| **GCH1** | rs3783641 | C__25800745_10 |
|  | rs4411417 | C__11164699_10 |
|  | rs1050450 | Self designed |
|  | rs6311 | C___8695278_10 |
| **GPX1** | rs6313 | C___3042197_1_ |
| **HTR2A** | rs6323 | Self designed |
|  | rs1137070 | C___8878813_20 |
| **MAOA** | rs1801133 | C___1202883_20 |
|  | rs11127292 | C___8971225_10 |
| **MTHFR** | rs1799983 | C___3219460_20 |
| **MYT1L** | rs1799971 | Self designed |
| **NOS3** | rs2230912 | C__15853715_20 |
| **OPRM1** | rs2522833 | C___2553139_10 |
| **P2RX7** | rs573542 | C____903247_10 |
| **PCLO** | rs4371369 | C____372246_20 |
| **SCN9A** | rs4387806 | C__27943991_10 |
|  | rs4453709 | C____259382_20 |
|  | rs4597545 | C____518820_10 |
|  | rs6746030 | C__29330435_10 |
|  | rs6754031 | C__29108389_10 |
|  | rs7607967 | C____372249_10 |
|  | rs12620053 | C__31157449_10 |
|  | rs12994338 | C__30668947_10 |
|  | rs13017637 | C__30668948_10 |
|  | rs28929474 | C__34508510_10 |
|  | rs25531 | Self designed |
| **SERPINA1** | rs25532 | Self designed |
| **SLC6A4** | rs4880 | C___8709053_10 |
|  | rs3746544 | C__27494002_10 |
| **SOD2** | rs8192619 | C__25961904_10 |
| **SNAP25** | rs3771863 | C__27498949_10 |
| **TAAR1** | rs4964728 | C__31582257_20 |
| **TACR1** | rs7310505 | C__29227804_10 |
| **TXNRD1** |  |  |
|  |  |  |

SNP, single nucleotide polymorphism.

Acronyms of genes as follows: ADRA1A, adrenoceptor alpha 1A; ADRB2, adrenoceptor beta 2; ADRB3, adrenoceptor beta 3; APOE, apolipoprotein E; BDNF-AS, brain-derived neurotrophic factor antisense RNA; CNR1, cannabinoid receptor 1; COMT, catechol-O-methyltransferase; CREB1, CAMP responsive element binding protein 1; CRHR1, corticotrophin-releasing hormone receptor 1; DRD3, dopamine receptor D3; DRD4, dopamine receptor D4; FKBP5, FK506 Binding Protein 5; GABRB3, gamma-aminobutyric acid type A receptor beta 3 subunit; GBP1, guanylate binding protein 1; GCH1, GTP cyclohydrolase 1; GPX1, glutathione peroxidase 1; HTR2A, 5-hydroxytryptamine receptor 2A; MAOA, Monoamine oxidase A; MTHFR, methylenetetrahydrofolate reductase; MYT1L, myelin transcription factor 1 like; NOS3, nitric oxide synthase 3; OPRM1, opioid receptor μ1; P2RX7, purinergic receptor P2X 7; PCLO, piccolo presynaptic cytomatrix protein; SCN9A, sodium voltage-gated channel alpha subunit 9; SERPINA1, serpin family A member 1; SLC6A4, solute carrier family 6 member 4; SOD2, superoxide dismutase 2; SNAP25, synaptosome associated protein 25; TAAR1, trace amine associated receptor 1; TACR1, tachykinin receptor 1; TXNRD1, thioredoxin reductase 1.

Table S2. Rationale for the inclusion of the 64 single nucleotide polymorphisms (SNPs) in the present study

| **Gene**  SNP | **Previous studies on fibromyalgia susceptibility** | **Previous studies on fibromyalgia symptoms** | **Previous studies on fibromyalgia-related mechanisms or symptoms** |
| --- | --- | --- | --- |
| **ADRA1A** |  |  |  |
| rs574584 | [1] | [1] |  |
| rs1048101 | [1] | [1] |  |
| rs1383914 | [1] | [1] |  |
| **ADRB2** |  |  |  |
| rs1042713 | [1] | [1] |  |
| rs1042714 | [1] | [1] |  |
| **ADRB3** |  |  |  |
| rs4994 | [1] | [1] |  |
| **APOE** |  |  |  |
| rs429358 |  |  | Alzheimer's Disease[2]  Dementia [3]  Cognitive performance[4] |
| **BDNF-AS** |  |  |  |
| rs6265 |  |  | Major depression[5] |
| rs7124442 |  |  | Major depression [6] |
| **CNR1** |  |  |  |
| rs806377 |  |  | Happiness[7] |
| **COMT** |  |  |  |
| rs4633 | [8–10] | [9,10] |  |
| rs6269 | [9] | [8,9] |  |
| rs165599 | [9,10] | [9,10] |  |
| rs2097903 | [9] | [9] |  |
| **CREB1** |  |  |  |
| rs2254137 |  |  | Cognitive performance [11] |
| **CRHR1** |  |  |  |
| rs242940 |  |  | Resilience[12] |
| rs7209436 |  |  | Resilience[13] |
| **DRD3** |  |  |  |
| rs6280 | [14] | [14] |  |
| **DRD4** |  |  |  |
| rs1800443 |  |  | Response to drug therapy[15] |
| rs1800955 |  |  | Personality [16] |
| **FKBP5** |  |  |  |
| rs1360780 |  |  | Resilience and vulnerability[17] |
| rs3800373 |  |  | Resilience and vulnerability[17] |
| rs9296158 |  |  | Resilience and vulnerability [18] |
| rs9470080 |  |  | Resilience and vulnerability [17] |
| **GABRB3** |  |  |  |
| rs4906902 | [19] |  |  |
| **GBP1** |  |  |  |
| rs7911 | [19] |  |  |
| **GCH1** |  |  |  |
| rs841 | [20] |  |  |
| rs752688 | [20] |  |  |
| rs3783641 | [20] |  |  |
| rs4411417 | [20] |  |  |
| **GPX1** |  |  |  |
| rs1050450 |  |  | Oxidative stress[21] |
| **HTR2A** |  |  |  |
| rs6311 | [22] |  |  |
| rs6313 | [22] |  |  |
| **MAOA** |  |  |  |
| rs6323 |  |  | Major depression[23] |
| rs1137070 |  |  | Major depression[23] |
| **MTHFR** |  |  |  |
| rs1801133 | [24] |  |  |
| **MYT1L** |  |  |  |
| rs11127292 | [25] |  |  |
| **NOS3** |  |  |  |
| rs1799983 |  |  | Cardiovascular risk[26] |
| **OPRM1** |  |  |  |
| rs1799971 |  | [27] |  |
| **P2RX7** |  |  |  |
| rs2230912 |  |  | Mood disorders [28] |
| **PCLO** |  |  |  |
| rs2522833 |  |  | Major depression[29] |
| **SCN9A** |  |  |  |
| rs573542 | [1] | [1] |  |
| rs4371369 | [30] | [30] |  |
| rs4387806 | [30] | [30] |  |
| rs4453709 | [30] | [30] |  |
| rs4597545 | [30] | [30] |  |
| rs6746030 | [30] | [30] |  |
| rs6754031 | [30] | [30] |  |
| rs7607967 | [30] | [30] |  |
| rs12620053 | [30] | [30] |  |
| rs12994338 | [30] | [30] |  |
| rs13017637 | [30] | [30] |  |
| **SERPINA1** |  |  |  |
| rs28929474 |  |  | Granulomatosis with polyangiitis[31] |
| **SLC6A4** |  |  |  |
| rs25531 | [32,33] | [33] |  |
| rs25532 | [32,33] | [33] |  |
| **SOD2** |  |  |  |
| rs4880 |  |  | Oxidative stress [34] |
| **SNAP25** |  |  |  |
| rs3746544 | [35] | [35] |  |
| **TAAR1** |  |  |  |
| rs8192619 | [19] |  |  |
| **TACR1** |  |  |  |
| rs3771863 | [36] |  |  |
| **TXNRD1** |  |  |  |
| rs4964728 |  |  | Longevity [37] |
| rs7310505 |  |  | Longevity[37] |

Acronyms of genes as follows: ADRA1A, adrenoceptor alpha 1A; ADRB2, adrenoceptor beta 2; ADRB3, adrenoceptor beta 3; APOE, apolipoproteine E; BDNF-AS, brain-derived neurotrophic factor antisense RNA; CNR1, cannabinoid receptor 1; COMT, catechol-O-methyltransferase; CREB1, CAMP responsive element binding protein 1; CRHR1, corticotrophin-releasing hormone receptor 1; DRD3, dopamine receptor D3; DRD4, dopamine receptor D4; FKBP5, FK506 Binding Protein 5; GABRB3, gamma-aminobutyric acid type A receptor beta 3 subunit; GBP1, guanylate binding protein 1; GCH1, GTP cyclohydrolase 1; GPX1, glutathione peroxidase 1; HTR2A, 5-hydroxytryptamine receptor 2A; MAOA, Monoamine oxidase A; MTHFR, methylene tetrahydrofolatereductase; MYT1L, myelin transcription factor 1 like; NOS3, nitric oxide synthase 3; OPRM1, opioid receptor μ1; P2RX7, purinergic receptor P2X 7; PCLO, piccolo presynaptic cytomatrixprotein; SCN9A, sodium voltage-gated channel alpha subunit 9; SERPINA1, serpin family A member 1; SLC6A4, solute carrier family 6 member 4; SOD2, superoxide dismutase 2; SNAP25, synaptosome associated protein 25; TAAR1, trace amine associated receptor 1; TACR1, tachykinin receptor 1; TXNRD1, thioredoxinreductase 1.

**REFERENCES**

1. Vargas-Alarcón G, Fragoso JM, Cruz-Robles D, Vargas A, Martinez A, Lao-Villadóniga JI, et al. Association of adrenergic receptor gene polymorphisms with different fibromyalgia syndrome domains. Arthritis Rheum. 2009;60:2169–73.

2. Urfer-Buchwalder A, Urfer R. Identification of a Nuclear Respiratory Factor 1 Recognition Motif in the Apolipoprotein E Variant APOE4 linked to Alzheimer’s Disease. Sci. Rep. [Internet]. 2017;7:40668. Available from: http://www.ncbi.nlm.nih.gov/pubmed/28094792

3. Skrobot OA, McKnight AJ, Passmore PA, Seripa D, Mecocci P, Panza F, et al. A Validation Study of Vascular Cognitive Impairment Genetics Meta-Analysis Findings in an Independent Collaborative Cohort. J. Alzheimers. Dis. [Internet]. 2016;53:981–9. Available from: http://www.ncbi.nlm.nih.gov/pubmed/27314523

4. Zhen J, Huang X, Van Halm-Lutterodt N, Dong S, Ma W, Xiao R, et al. ApoE rs429358 and rs7412 Polymorphism and Gender Differences of Serum Lipid Profile and Cognition in Aging Chinese Population. Front. Aging Neurosci. [Internet]. 2017;9:248. Available from: http://www.ncbi.nlm.nih.gov/pubmed/28824412

5. Taylor MK, Beckerley SE, Henniger NE, Hernández LM, Larson GE, Granger DA. A genetic risk factor for major depression and suicidal ideation is mitigated by physical activity. Psychiatry Res. [Internet]. Elsevier; 2017;249:304–6. Available from: http://dx.doi.org/10.1016/j.psychres.2017.01.002

6. Zhang K, Yang C, Xu Y, Sun N, Yang H, Liu J, et al. Genetic association of the interaction between the BDNF and GSK3B genes and major depressive disorder in a Chinese population. J. Neural Transm. [Internet]. 2010;117:393–401. Available from: http://www.ncbi.nlm.nih.gov/pubmed/20033742

7. Matsunaga M, Isowa T, Yamakawa K, Fukuyama S, Shinoda J, Yamada J, et al. Genetic variations in the human cannabinoid receptor gene are associated with happiness. PLoS One [Internet]. 2014;9:e93771. Available from: http://www.ncbi.nlm.nih.gov/pubmed/24690898

8. Martínez-Jauand M, Sitges C, Rodríguez V, Picornell a., Ramon M, Buskila D, et al. Pain sensitivity in fibromyalgia is associated with catechol-O- methyltransferase (COMT) gene. Eur. J. Pain (United Kingdom). 2013;17:16–27.

9. Vargas-Alarcón G, Fragoso J-M, Cruz-Robles D, Vargas A, Vargas A, Lao-Villadóniga J-I, et al. Catechol-O-methyltransferase gene haplotypes in Mexican and Spanish patients with fibromyalgia. Arthritis Res. Ther. 2007;9:R110.

10. Park DJ, Kim SH, Nah SS, Lee JH, Kim SK, Lee YA, et al. Association between catechol-O-methyl transferase gene polymorphisms and fibromyalgia in a Korean population: A case-control study. Eur. J. Pain [Internet]. 2016;20:1131–9. Available from: http://www.ncbi.nlm.nih.gov/pubmed/26949490

11. Guo J, Liu Z, Dai H, Zhu Z, Wang H, Yang C, et al. Preliminary investigation of the influence of CREB1 gene polymorphisms on cognitive dysfunction in Chinese patients with major depression. Int. J. Neurosci. [Internet]. 2014;124:22–9. Available from: http://www.ncbi.nlm.nih.gov/pubmed/23844928

12. Feder A, Nestler EJ, Charney DS. Psychobiology and molecular genetics of resilience. Nat. Rev. Neurosci. [Internet]. Nature Publishing Group; 2009;10:446–57. Available from: http://www.nature.com/doifinder/10.1038/nrn2649

13. Arnsten AFT. Stress signalling pathways that impair prefrontal cortex structure and function. Nat. Rev. Neurosci. [Internet]. Nature Publishing Group; 2009;10:410–22. Available from: http://www.nature.com/doifinder/10.1038/nrn2648

14. Potvin S, Larouche A, Normand E, de Souza JB, Gaumond I, Grignon S, et al. DRD3 Ser9Gly Polymorphism Is Related to Thermal Pain Perception and Modulation in Chronic Widespread Pain Patients and Healthy Controls. J. Pain. 2009;10:969–75.

15. Barbalic M, Schwartz GL, Chapman AB, Turner ST, Boerwinkle E. Kininogen gene (KNG) variation has a consistent effect on aldosterone response to antihypertensive drug therapy: the GERA study. Physiol. Genomics [Internet]. 2009 [cited 2017 Oct 4];39:56–60. Available from: http://www.ncbi.nlm.nih.gov/pubmed/19584173

16. Munafò MR, Yalcin B, Willis-Owen SA, Flint J. Association of the dopamine D4 receptor (DRD4) gene and approach-related personality traits: meta-analysis and new data. Biol. Psychiatry [Internet]. 2008;63:197–206. Available from: http://www.ncbi.nlm.nih.gov/pubmed/17574217

17. Wang Q, Shelton RC, Dwivedi Y. Interaction between early-life stress and FKBP5 gene variants in major depressive disorder and post-traumatic stress disorder: A systematic review and meta-analysis. J. Affect. Disord. [Internet]. 2017;225:422–8. Available from: http://www.ncbi.nlm.nih.gov/pubmed/28850857

18. Watkins LE, Han S, Harpaz-Rotem I, Mota NP, Southwick SM, Krystal JH, et al. FKBP5 polymorphisms, childhood abuse, and PTSD symptoms: Results from the National Health and Resilience in Veterans Study. Psychoneuroendocrinology [Internet]. 2016;69:98–105. Available from: http://www.ncbi.nlm.nih.gov/pubmed/27078785

19. Smith SB, Maixner DW, Fillingim RB, Slade G, Gracely RH, Ambrose K, et al. Large candidate gene association study reveals genetic risk factors and therapeutic targets for fibromyalgia. Arthritis Rheum. 2012;64:584–93.

20. Kim SK, Kim SH, Nah SS, Hyun Lee J, Hong SJ, Kim HS, et al. Association of guanosine triphosphate cyclohydrolase 1 gene polymorphisms with fibromyalgia syndrome in a Korean population. J. Rheumatol. 2013;40:316–22.

21. Bhatti P, Stewart PA, Hutchinson A, Rothman N, Linet MS, Inskip PD, et al. Lead exposure, polymorphisms in genes related to oxidative stress, and risk of adult brain tumors. Cancer Epidemiol. Biomarkers Prev. [Internet]. 2009;18:1841–8. Available from: http://www.ncbi.nlm.nih.gov/pubmed/19505917

22. Tander B, Gunes S, Boke O, Alayli G, Kara N, Bagci H, et al. Polymorphisms of the serotonin-2A receptor and catechol-O-methyltransferase genes: A study on fibromyalgia susceptibility. Rheumatol. Int. 2008;28:685–91.

23. Zhang J, Chen Y, Zhang K, Yang H, Sun Y, Fang Y, et al. A cis-phase interaction study of genetic variants within the MAOA gene in major depressive disorder. Biol. Psychiatry [Internet]. Elsevier Inc.; 2010;68:795–800. Available from: http://dx.doi.org/10.1016/j.biopsych.2010.06.004

24. Inanir A, Yigit S, Tekcan A, Pinarli FA, Inanir S, Karakus N. Angiotensin converting enzyme and methylenetetrahydrofolate reductase gene variations in fibromyalgia syndrome. Gene [Internet]. Elsevier B.V.; 2015; Available from: http://linkinghub.elsevier.com/retrieve/pii/S0378111915003571

25. Docampo E, Escaramís G, Gratacòs M, Villatoro S, Puig A, Kogevinas M, et al. Genome-wide analysis of single nucleotide polymorphisms and copy number variants in fibromyalgia suggest a role for the central nervous system. Pain [Internet]. International Association for the Study of Pain; 2014;155:1102–9. Available from: http://dx.doi.org/10.1016/j.pain.2014.02.016

26. Pal GK, Adithan C, Umamaheswaran G, Pal P, Nanda N, Indumathy J, et al. Endothelial nitric oxide synthase gene polymorphisms are associated with cardiovascular risks in prehypertensives. J. Am. Soc. Hypertens. [Internet]. 2016;10:865–72. Available from: http://www.ncbi.nlm.nih.gov/pubmed/27697448

27. Tour J, Löfgren M, Mannerkorpi K, Gerdle B, Larsson A, Palstam A, et al. Gene-to-gene interactions regulate endogenous pain modulation in fibromyalgia patients and healthy controls—antagonistic effects between opioid and serotonin-related genes. Pain [Internet]. 2017;0:1. Available from: http://insights.ovid.com/crossref?an=00006396-900000000-99287

28. Soronen P, Mantere O, Melartin T, Suominen K, Vuorilehto M, Rytsälä H, et al. P2RX7 gene is associated consistently with mood disorders and predicts clinical outcome in three clinical cohorts. Am. J. Med. Genet. B. Neuropsychiatr. Genet. [Internet]. 2011;156B:435–47. Available from: http://www.ncbi.nlm.nih.gov/pubmed/21438144

29. Sullivan PF, de Geus EJC, Willemsen G, James MR, Smit JH, Zandbelt T, et al. Genome-wide association for major depressive disorder: a possible role for the presynaptic protein piccolo. Mol. Psychiatry [Internet]. 2009;14:359–75. Available from: http://www.ncbi.nlm.nih.gov/pubmed/19065144

30. Vargas-Alarcon G, Alvarez-Leon E, Fragoso J-M, Vargas A, Martinez A, Vallejo M, et al. A SCN9A gene-encoded dorsal root ganglia sodium channel polymorphism associated with severe fibromyalgia. BMC Musculoskelet. Disord. [Internet]. BioMed Central Ltd; 2012;13:23. Available from: http://www.biomedcentral.com/1471-2474/13/23

31. Borgmann S, Endisch G, Urban S, Sitter T, Fricke H. A linkage disequilibrium between genes at the serine protease inhibitor gene cluster on chromosome 14q32.1 is associated with Wegener’s granulomatosis. Clin. Immunol. [Internet]. 2001 [cited 2017 Oct 4];98:244–8. Available from: http://linkinghub.elsevier.com/retrieve/pii/S1521661600949623

32. Gursoy S. Absence of association of the serotonin transporter gene polymorphism with the mentally healthy subset of fibromyalgia patients. Clin. Rheumatol. 2002;21:194–7.

33. Offenbaecher M, Bondy B, de Jonge S, Glatzeder K, Krüger M, Schoeps P, et al. Possible association of fibromyalgia with a polymorphism in the serotonin transporter gene regulatory region. Arthritis Rheum. 1999;42:2482–8.

34. Kim JH, Lee M-R, Hong Y-C. Modification of the association of bisphenol A with abnormal liver function by polymorphisms of oxidative stress-related genes. Environ. Res. [Internet]. 2016;147:324–30. Available from: http://www.ncbi.nlm.nih.gov/pubmed/26922413

35. Balkarli A, Sengül C, Tepeli E, Balkarli H, Cobankara V. Synaptosomal-associated protein 25 (Snap-25) gene Polymorphism frequency in fibromyalgia syndrome and relationship with clinical symptoms. BMC Musculoskelet. Disord. [Internet]. 2014;15:191. Available from: http://www.biomedcentral.com/1471-2474/15/191

36. Rodriguez-Rodriguez L, Ramón Lamas J, Abásolo L, Baena S, Olano-Martin E, Collado A, et al. The rs3771863 single nucleotide polymorphism of the TACR1 gene is associated to a lower risk of sicca syndrome in fibromyalgia patients. Clin. Exp. Rheumatol. [Internet]. 2015 [cited 2015 Apr 14];33:33–40. Available from: http://www.ncbi.nlm.nih.gov/pubmed/25786041

37. Dato S, De Rango F, Crocco P, Passarino G, Rose G. Antioxidants and Quality of Aging: Further Evidences for a Major Role of TXNRD1 Gene Variability on Physical Performance at Old Age. Oxid. Med. Cell. Longev. [Internet]. 2015;2015:926067. Available from: http://www.ncbi.nlm.nih.gov/pubmed/26064428

Table S3. Thermal cycling conditions

|  | **AmpliTaq Gold® enzyme activation** | **PCR** |
| --- | --- | --- |
|  | HOLD CYCLE | (40 cycles)  Denature - Anneal/Extend |
| **Time** | 10 min | 15 sec -1 min |
| **Temperature** | 95 °C | 92 °C-60 °C |

PCR, polymerase chain reaction

Table S4. Further details of the single nucleotide polymorphisms (SNP) included in the present study

| **SNP** | **Chromosome details** | **Biological function** | **cDNA position** | **Protein position** | **Aa** | **SIFT** | **Polymorphism Phenotype** | **Clinical significance** |
| --- | --- | --- | --- | --- | --- | --- | --- | --- |
| **ADRA1A** |  |  |  |  |  |  |  |  |
| rs574584 | 8:26866167-26866167 | Upstream gene variant |  |  |  |  |  |  |
| rs1048101 | 8:26770511-26770511 | Missense | 1475 | 347 | C/R | Tolerated  (0.71) | Benign  (0) |  |
| rs1383914 | 8:26865532-26865532 | Upstream gene variant |  |  |  |  |  |  |
| **ADRB2** |  |  |  |  |  |  |  |  |
| rs1042713 | 5:148826877-148826877 | Missense | 1633 | 16 | G/R | Tolerated  (0.17) | Benign  (0.143) | Drug response,  Risk factor |
| rs1042714 | 5:148826910-148826910 | Missense | 1666 | 27 | E/Q | Tolerated  (0.47) | Benign  (0.008) | Risk factor, |
| **ADRB3** |  |  |  |  |  |  |  |  |
| rs4994 | 8:37966280-37966280 | Missense | 686 | 64 | W/R | Tolerated  (1) | Benign  (0) | Risk factor |
| **APOE** |  |  |  |  |  |  |  |  |
| rs429358 | 19:44908684-44908684 | Missense | 499 | 130 | C/R | Tolerated  (1) | Benign  (0) | Pathogenic,  Association |
| **BDNF-AS** |  |  |  |  |  |  |  |  |
| rs6265 | 11:27658369-27658369 | Missense  Val66Met | 864 | 74 | V/M | Tolerated  (0.2) | Possibly damaging  (0.791) | Benign,  Risk factor,  Protective |
| rs7124442 | 11:27655494-27655494 | 3 prime UTR variant |  |  |  |  |  |  |
| **CNR1** |  |  |  |  |  |  |  |  |
| rs806377 | 6:88149004-88149004 | Upstream gene variant |  |  |  |  |  |  |
| **COMT** |  |  |  |  |  |  |  |  |
| rs4633 | 22:19962712-19962712 | Synonymous,NMD transcript | 351 | 62 | H |  |  | Benign |
| rs6269 | 22:19962429-19962429 | Intron variant, NMD transcript variant |  |  |  |  |  |  |
| rs165599 | 22:19969258-19969258 | Downstream gene variant |  |  |  |  |  |  |
| rs2097903 | 7:10642782-10642782 | Intron variant, Non coding transcript variant |  |  |  |  |  |  |
| **CREB1** |  |  |  |  |  |  |  |  |
| rs2254137 | 2:207579304-207579304 | Intron variant |  |  |  |  |  |  |
| **CRHR1** |  |  |  |  |  |  |  |  |
| rs242940 | 17:45815234-45815234 | Intron variant |  |  |  |  |  |  |
| rs7209436 | 17:45792776-45792776 | Intron variant |  |  |  |  |  |  |
| **DRD3** |  |  |  |  |  |  |  |  |
| rs6280 | 3:114171968-114171968 | Missense | 456 | 9 | G/S | Tolerated low confidence (1) | Benign  (0.003) |  |
| **DRD4** |  |  |  |  |  |  |  |  |
| rs1800443 | 11:639830-639830 | Missense | 593 | 194 | V/G | Deleterious(0) | Unknown  (0) | Benign |
| rs1800955 | 11:636784-636784 | Upstream gene variant |  |  |  |  |  |  |
| **FKBP5** |  |  |  |  |  |  |  |  |
| rs1360780 | 6:35639794-35639794 | Intron variant |  |  |  |  |  |  |
| rs3800373 | 6:35574699-35574699 | 3 prime UTR variant |  |  |  |  |  |  |
| rs9296158 | 6:35599305-35599305 | Intron variant |  |  |  |  |  |  |
| rs9470080 | 6:35678658-35678658 | Intron variant |  |  |  |  |  |  |
| **GABRB3** |  |  |  |  |  |  |  |  |
| rs4906902 | 15:26774621-26774621 | Upstream gene variant |  |  |  |  |  |  |
| **GBP1** |  |  |  |  |  |  |  |  |
| rs7911 | 1:89052437-89052437 | 3 prime UTR variant |  |  |  |  |  |  |
| **GCH1** |  |  |  |  |  |  |  |  |
| rs841 | 14:54843774-54843774 | Non coding transcript exon variant, Non coding transcript variant |  |  |  |  |  |  |
| rs752688 | 14:54844851-54844851 | Intron variant, Non coding transcript variant |  |  |  |  |  |  |
| rs3783641 | 14:54893421-54893421 | Intron variant, Non coding transcript variant |  |  |  |  |  |  |
| rs4411417 | 14:54853845-54853845 | Intron variant, Non coding transcript variant |  |  |  |  |  |  |
| **GPX1** |  |  |  |  |  |  |  |  |
| rs1050450 | 3:49357401-49357401 | Downstream gene variant |  |  |  |  |  |  |
| **HTR2A** |  |  |  |  |  |  |  |  |
| rs6311 | 13:46897343-46897343 | Upstream gene variant |  |  |  |  |  |  |
| rs6313 | 13:46895805-46895805 | Synonymous | 234 | 34 | S |  |  |  |
| **MAOA** |  |  |  |  |  |  |  |  |
| rs6323 | X:43731789-43731789 | Synonymous | 1014 | 297 | R |  |  |  |
| rs1137070 | X:43744144-43744144 | Synonymous | 1533 | 470 | D |  |  |  |
| **MTHFR** |  |  |  |  |  |  |  |  |
| rs1801133 | 1:11796321-11796321 | Missense | 788 | 263 | A/V | Deleterious  (0.02) | Probably Damaging  (0.999) | Uncertain significance,  Not provided,  Benign,  Drug response |
| **MYT1L** |  |  |  |  |  |  |  |  |
| rs11127292 | 2:2026171-2026171 | Intron variant |  |  |  |  |  |  |
| **NOS3** |  |  |  |  |  |  |  |  |
| rs1799983 | 7:150999023-150999023 | Missense | 1251 | 298 | D/E | Tolerated (1) | Benign  (0.001) | Pathogenic,  Risk factor |
| **OPRM1** |  |  |  |  |  |  |  |  |
| rs1799971 | 6:154039662 | Missense | 118A>G | Asn40Asp | Tolerated (0.05) | Benign (0.138) |  |  |
| **P2RX7** |  |  |  |  |  |  |  |  |
| rs2230912 | 12:121184393-121184393 | 3_prime_UTR_variant,NMD_transcript_variant | 1323 | 460 | Q/R |  |  |  |
| **PCLO** |  |  |  |  |  |  |  |  |
| rs2522833 | 7:82824392-82824392 | Missense | 14778 | 4814 | S/A | Tolerated (0.54) | Unknown (0) |  |
| **SCN9A** |  |  |  |  |  |  |  |  |
| rs573542 | 8:26866301-26866301 | Upstream gene variant |  |  |  |  |  |  |
| rs4371369 | 2:166260145-166260145 | Intron variant |  |  |  |  |  |  |
| rs4387806 | 2:166294304-166294304 | Intron variant |  |  |  |  |  |  |
| rs4453709 | 2:166269944-166269944 | Intron variant, Non coding transcript variant |  |  |  |  |  |  |
| rs4597545 | 2:166293988-166293988 | Intron variant |  |  |  |  |  |  |
| rs6746030 | 2:166242648-166242648 | Missense | 3822 | 1161 | W/R | Tolerated (1) | Benign (0) |  |
| rs6754031 | 2:166298928-166298928 | Intron variant |  |  |  |  |  |  |
| rs7607967 | 2:166256826-166256826 | Intron variant |  |  |  |  |  |  |
| rs12620053 | 2:166301776-166301776 | Intron variant |  |  |  |  |  |  |
| rs12994338 | 2:166303519-166303519 | Intron variant |  |  |  |  |  |  |
| rs13017637 | 2:166303436-166303436 | Intron variant |  |  |  |  |  |  |
| **SERPINA1** |  |  |  |  |  |  |  |  |
| rs28929474 | 14:94378610-94378610 | Missense | 1373 | 366 | E/K | Tolerated (0.07) | Probably damaging (0.997) | Pathogenic,  Other |
| **SLC6A4** |  |  |  |  |  |  |  |  |
| rs25531 | 17:30237328-30237328 | Coding for the serotonin transporter. Upstream gene variant |  |  |  |  |  |  |
| rs25532 | 17:30237152-30237152 | Upstream gene variant |  |  |  |  |  |  |
| **SOD2** |  |  |  |  |  |  |  |  |
| rs4880 | 6:159692840-159692840 | Missense | 158 | 16 | V/A | Tolerated (0.93) | Benign (0) | Benign,  Drug Response,  Risk factor |
| **SNAP25** |  |  |  |  |  |  |  |  |
| rs3746544 | 20:10306436-10306436 | 3 prime UTR variant |  |  |  |  |  |  |
| **TAAR1** |  |  |  |  |  |  |  |  |
| rs8192619 | 6:132645209-132645209 | Synonymous | 795 | 265 | C |  |  |  |
| **TACR1** |  |  |  |  |  |  |  |  |
| rs3771863 | 2:75192588-75192588 | Intron variant |  |  |  |  |  |  |
| **TXNRD1** |  |  |  |  |  |  |  |  |
| rs4964728 | 12:104255955-104255955 | Intron variant |  |  |  |  |  |  |
| rs7310505 | 12:104260770-104260770 | Intron variant |  |  |  |  |  |  |

Aa, amino acid.

Acronyms of genes as follows: ADRA1A, adrenoceptor alpha 1A; ADRB2, adrenoceptor beta 2; ADRB3, adrenoceptor beta 3; APOE, apolipoprotein E; BDNF-AS, brain-derived neurotrophic factor antisense RNA; CNR1, cannabinoid receptor 1; COMT, catechol-O-methyltransferase; CREB1, CAMP responsive element binding protein 1; CRHR1, corticotrophin-releasing hormone receptor 1; DRD3, dopamine receptor D3; DRD4, dopamine receptor D4; FKBP5, FK506 Binding Protein 5; GABRB3, gamma-aminobutyric acid type A receptor beta 3 subunit; GBP1, guanylate binding protein 1; GCH1, GTP cyclohydrolase 1; GPX1, glutathione peroxidase 1; HTR2A, 5-hydroxytryptamine receptor 2A; MAOA, Monoamine oxidase A; MTHFR, methylenetetrahydrofolate reductase; MYT1L, myelin transcription factor 1 like; NOS3, nitric oxide synthase 3; OPRM1, opioid receptor μ1; P2RX7, purinergic receptor P2X 7; PCLO, piccolo presynaptic cytomatrix protein; SCN9A, sodium voltage-gated channel alpha subunit 9; SERPINA1, serpin family A member 1; SLC6A4, solute carrier family 6 member 4; SOD2, superoxide dismutase 2; SNAP25, synaptosome associated protein 25; TAAR1, trace amine associated receptor 1; TACR1, tachykinin receptor 1; TXNRD1, thioredoxin reductase 1.

Table S5. Socio-demographic and clinical characteristics of the study samples

|  | | Fibromyalgia (*n*=314) | | | | Controls  (*n*=112) | | | |
| --- | --- | --- | --- | --- | --- | --- | --- | --- | --- |
| Age, mean (*SD*), years old | | 52.3 | ( | 8.7 | ) | 48.2 | ( | 7.6 | ) |
| Education level, *n (%)* | |  |  |  |  |  |  |  |  |
|  | Unfinished studies | 33 | ( | 10.5 | ) | 6 | ( | 5.4 | ) |
|  | Primary | 157 | ( | 50.0 | ) | 32 | ( | 28.5 | ) |
|  | Secondary (and vocational) | 88 | ( | 28.0 | ) | 48 | ( | 42.9 | ) |
|  | University | 36 | ( | 11.5 | ) | 26 | ( | 23.2 | ) |
| Marital status*, n (%)* | |  |  |  |  |  |  |  |  |
|  | Married | 246 | ( | 78.3 | ) | 84 | ( | 75.0 | ) |
|  | Single | 24 | ( | 7.7 | ) | 11 | ( | 9.8 | ) |
|  | Separated/divorced | 29 | ( | 9.2 | ) | 13 | ( | 11.6 | ) |
|  | Widow | 15 | ( | 4.8 | ) | 3 | ( | 2.7 | ) |
|  | Missing data | 0 | ( | 0.0 | ) | 1 | ( | 0.9 | ) |
| Working status, *n (%)* | |  |  |  |  |  |  |  |  |
|  | Working | 82 | ( | 26.1 | ) | 49 | ( | 43.8 | ) |
|  | Household | 105 | ( | 33.5 | ) | 38 | ( | 33.9 | ) |
|  | Incapacity pension or sick leave | 63 | ( | 20.1 | ) | 4 | ( | 3.5 | ) |
|  | Unemployed | 51 | ( | 16.2 | ) | 14 | ( | 12.5 | ) |
|  | Others | 13 | ( | 4.1 | ) | 7 | ( | 6.3 | ) |
| Tender points count, mean (*SD*) | | 16.9 | ( | 1.8 | ) | 2.8 | ( | 3.0 | ) |

*SD*, Standard Deviation.

Table S6. Genotype frequencies of single nucleotide polymorphisms (SNP) in fibromyalgia (FM) and non-fibromyalgia (controls, HC) women

| **Gene** | **SNP** | **Genotype** | **FM,**  ***n*** | **(** | **%** | **)** | **HC, *n*** | **(** | **%** | **)** | **χ^2^** | ***P*** |
| --- | --- | --- | --- | --- | --- | --- | --- | --- | --- | --- | --- | --- |
| **ADRA1A** |  |  |  |  |  |  |  |  |  |  |  |  |
|  | rs574584 | CC | 4 | ( | 1.3 | ) | 0 | ( | 0.0 | ) | 1.45 | 0.229 |
|  |  | CT/TT | 309 | ( | 98.7 | ) | 112 | ( | 100.0 | ) |  |  |
|  |  | C | 40 | ( | 6.4 | ) | 16 | ( | 7.1 | ) | 0.15 | 0.697 |
|  |  | T | 586 | ( | 93.6 | ) | 208 | ( | 92.9 | ) |  |  |
|  | rs1048101 | A | 78 | ( | 25.2 | ) | 27 | ( | 24.3 | ) | 0.10 | 0.952 |
|  |  | AG | 165 | ( | 53.2 | ) | 61 | ( | 55.0 | ) |  |  |
|  |  | G | 67 | ( | 21.6 | ) | 23 | ( | 20.7 | ) |  |  |
|  |  | A | 321 | ( | 51.8 | ) | 115 | ( | 51.8 | ) | <0.01 | 0.994 |
|  |  | G | 299 | ( | 48.2 | ) | 107 | ( | 48.2 | ) |  |  |
|  | rs1383914 | C | 78 | ( | 25.2 | ) | 25 | ( | 22.5 | ) | 2.49 | 0.287 |
|  |  | CT | 135 | ( | 43.7 | ) | 58 | ( | 52.3 | ) |  |  |
|  |  | TT | 96 | ( | 31.1 | ) | 28 | ( | 25.2 | ) |  |  |
|  |  | C | 291 | ( | 47.1 | ) | 108 | ( | 48.6 | ) | 0.16 | 0.689 |
|  |  | T | 327 | ( | 52.9 | ) | 114 | ( | 51.4 | ) |  |  |
| **ADRB2** |  |  |  |  |  |  |  |  |  |  |  |  |
|  | rs1042713 | A | 56 | ( | 18.0 | ) | 16 | ( | 14.5 | ) | 1.68 | 0.431 |
|  |  | AG | 148 | ( | 47.6 | ) | 49 | ( | 44.5 | ) |  |  |
|  |  | G | 107 | ( | 34.4 | ) | 45 | ( | 40.9 | ) |  |  |
|  |  | A | 260 | ( | 41.8 | ) | 81 | ( | 36.8 | ) | 1.67 | 0.196 |
|  |  | G | 362 | ( | 58.2 | ) | 139 | ( | 63.2 | ) |  |  |
|  | rs1042714 | C | 118 | ( | 38.1 | ) | 39 | ( | 35.1 | ) | 0.55 | 0.758 |
|  |  | CG | 147 | ( | 47.4 | ) | 53 | ( | 47.7 | ) |  |  |
|  |  | G | 45 | ( | 14.5 | ) | 19 | ( | 17.1 | ) |  |  |
|  |  | C | 383 | ( | 61.8 | ) | 131 | ( | 59.0 | ) | 0.53 | 0.468 |
|  |  | G | 237 | ( | 38.2 | ) | 91 | ( | 41.0 | ) |  |  |
| **ADRB3** |  |  |  |  |  |  |  |  |  |  |  |  |
|  | rs4994 | A | 267 | ( | 85.0 | ) | 95 | ( | 84.8 | ) | 0.59 | 0.745 |
|  |  | AG | 46 | ( | 14.6 | ) | 16 | ( | 14.3 | ) |  |  |
|  |  | G | 1 | ( | 0.3 | ) | 1 | ( | 0.9 | ) |  |  |
|  |  | A | 580 | ( | 92.4 | ) | 206 | ( | 92.0 | ) | 0.04 | 0.850 |
|  |  | G | 48 | ( | 7.6 | ) | 18 | ( | 8.0 | ) |  |  |
| **APOE** |  |  |  |  |  |  |  |  |  |  |  |  |
|  | rs429358 | C | 4 | ( | 1.3 | ) | 3 | ( | 2.7 | ) | 2.22 | 0.330 |
|  |  | CT | 53 | ( | 17.0 | ) | 24 | ( | 21.4 | ) |  |  |
|  |  | TT | 255 | ( | 81.7 | ) | 85 | ( | 75.9 | ) |  |  |
|  |  | C | 61 | ( | 9.8 | ) | 30 | ( | 13.4 | ) | 2.25 | 0.133 |
|  |  | T | 563 | ( | 90.2 | ) | 194 | ( | 86.6 | ) |  |  |
| **BDNF-AS** |  |  |  |  |  |  |  |  |  |  |  |  |
|  | rs6265 | C | 187 | ( | 60.1 | ) | 72 | ( | 64.9 | ) | 0.87 | 0.649 |
|  |  | CT | 109 | ( | 35.0 | ) | 35 | ( | 31.5 | ) |  |  |
|  |  | TT | 15 | ( | 4.8 | ) | 4 | ( | 3.6 | ) |  |  |
|  |  | C | 483 | ( | 77.7 | ) | 179 | ( | 80.6 | ) | 0.86 | 0.354 |
|  |  | T | 139 | ( | 22.3 | ) | 43 | ( | 19.4 | ) |  |  |
| **COMT** |  |  |  |  |  |  |  |  |  |  |  |  |
|  | rs4633 | C | 81 | ( | 25.9 | ) | 35 | ( | 31.3 | ) | 1.56 | 0.459 |
|  |  | CT | 154 | ( | 49.2 | ) | 54 | ( | 48.2 | ) |  |  |
|  |  | TT | 78 | ( | 24.9 | ) | 23 | ( | 20.5 | ) |  |  |
|  |  | C | 316 | ( | 50.5 | ) | 124 | ( | 55.4 | ) | 1.57 | 0.210 |
|  |  | T | 310 | ( | 49.5 | ) | 100 | ( | 44.6 | ) |  |  |
|  | rs6269 | A | 98 | ( | 31.5 | ) | 38 | ( | 34.2 | ) | 2.31 | 0.315 |
|  |  | AG | 156 | ( | 50.2 | ) | 47 | ( | 42.3 | ) |  |  |
|  |  | G | 57 | ( | 18.3 | ) | 26 | ( | 23.4 | ) |  |  |
|  |  | A | 352 | ( | 56.6 | ) | 123 | ( | 55.4 | ) | 0.09 | 0.760 |
|  |  | G | 270 | ( | 43.4 | ) | 99 | ( | 44.6 | ) |  |  |
|  | rs165599 | A | 157 | ( | 50.2 | ) | 48 | ( | 42.9 | ) | 2.33 | 0.312 |
|  |  | AG | 124 | ( | 39.6 | ) | 48 | ( | 42.9 | ) |  |  |
|  |  | G | 32 | ( | 10.2 | ) | 16 | ( | 14.3 | ) |  |  |
|  |  | A | 438 | ( | 70.0 | ) | 144 | ( | 64.3 | ) | 2.47 | 0.116 |
|  |  | G | 188 | ( | 30.0 | ) | 80 | ( | 35.7 | ) |  |  |
| **CREB1** |  |  |  |  |  |  |  |  |  |  |  |  |
|  | rs2254137 | A | 134 | ( | 43.1 | ) | 57 | ( | 51.4 | ) | 2.68 | 0.262 |
|  |  | AC | 145 | ( | 46.6 | ) | 42 | ( | 37.8 | ) |  |  |
|  |  | C | 32 | ( | 10.3 | ) | 12 | ( | 10.8 | ) |  |  |
|  |  | A | 413 | ( | 66.4 | ) | 156 | ( | 70.3 | ) | 1.12 | 0.291 |
|  |  | C | 209 | ( | 33.6 | ) | 66 | ( | 29.7 | ) |  |  |
| **CRHR1** |  |  |  |  |  |  |  |  |  |  |  |  |
|  | rs242940 | AA | 112 | ( | 35.9 | ) | 49 | ( | 44.1 | ) | 2.36 | 0.124 |
|  |  | AG/GG | 200 | ( | 64.1 | ) | 62 | ( | 55.9 | ) |  |  |
|  |  | A | 383 | ( | 61.4 | ) | 145 | ( | 65.3 | ) | 1.08 | 0.298 |
|  |  | G | 241 | ( | 38.6 | ) | 77 | ( | 34.7 | ) |  |  |
|  | rs7209436 | C | 131 | ( | 42.3 | ) | 57 | ( | 51.4 | ) | 2.90 | 0.234 |
|  |  | CT | 144 | ( | 46.5 | ) | 42 | ( | 37.8 | ) |  |  |
|  |  | TT | 35 | ( | 11.3 | ) | 12 | ( | 10.8 | ) |  |  |
|  |  | C | 406 | ( | 65.5 | ) | 156 | ( | 70.3 | ) | 1.69 | 0.194 |
|  |  | T | 214 | ( | 34.5 | ) | 66 | ( | 29.7 | ) |  |  |
| **DRD3** |  |  |  |  |  |  |  |  |  |  |  |  |
|  | rs6280 | C | 35 | ( | 11.1 | ) | 10 | ( | 8.9 | ) | 1.32 | 0.516 |
|  |  | CT | 141 | ( | 44.9 | ) | 46 | ( | 41.1 | ) |  |  |
|  |  | TT | 138 | ( | 43.9 | ) | 56 | ( | 50.0 | ) |  |  |
|  |  | C | 211 | ( | 33.6 | ) | 66 | ( | 29.5 | ) | 1.29 | 0.257 |
|  |  | T | 417 | ( | 66.4 | ) | 158 | ( | 70.5 | ) |  |  |
| **DRD4** |  |  |  |  |  |  |  |  |  |  |  |  |
|  | rs1800955 | C | 72 | ( | 24.2 | ) | 24 | ( | 22.2 | ) | 0.17 | 0.920 |
|  |  | CT | 142 | ( | 47.7 | ) | 53 | ( | 49.1 | ) |  |  |
|  |  | TT | 84 | ( | 28.2 | ) | 31 | ( | 28.7 | ) |  |  |
|  |  | C | 286 | ( | 48.0 | ) | 101 | ( | 46.8 | ) | 0.10 | 0.757 |
|  |  | T | 310 | ( | 52.0 | ) | 115 | ( | 53.2 | ) |  |  |
| **FKBP5** |  |  |  |  |  |  |  |  |  |  |  |  |
|  | rs3800373 | A | 163 | ( | 55.1 | ) | 53 | ( | 48.2 | ) | 1.69 | 0.430 |
|  |  | AC | 104 | ( | 35.1 | ) | 46 | ( | 41.8 | ) |  |  |
|  |  | C | 29 | ( | 9.8 | ) | 11 | ( | 10.0 | ) |  |  |
|  |  | A | 430 | ( | 72.6 | ) | 152 | ( | 69.1 | ) | 0.99 | 0.319 |
|  |  | C | 162 | ( | 27.4 | ) | 68 | ( | 30.9 | ) |  |  |
|  | rs9296158 | A | 29 | ( | 9.3 | ) | 10 | ( | 9.0 | ) | 0.42 | 0.810 |
|  |  | AG | 124 | ( | 39.7 | ) | 48 | ( | 43.2 | ) |  |  |
|  |  | G | 159 | ( | 51.0 | ) | 53 | ( | 47.7 | ) |  |  |
|  |  | A | 182 | ( | 29.2 | ) | 68 | ( | 30.6 | ) | 0.17 | 0.681 |
|  |  | G | 442 | ( | 70.8 | ) | 154 | ( | 69.4 | ) |  |  |
|  | rs9470080 | C | 134 | ( | 48.6 | ) | 50 | ( | 48.1 | ) | 0.65 | 0.722 |
|  |  | CT | 111 | ( | 40.2 | ) | 45 | ( | 43.3 | ) |  |  |
|  |  | TT | 31 | ( | 11.2 | ) | 9 | ( | 8.7 | ) |  |  |
|  |  | C | 379 | ( | 68.7 | ) | 145 | ( | 69.7 | ) | 0.08 | 0.780 |
|  |  | T | 173 | ( | 31.3 | ) | 63 | ( | 30.3 | ) |  |  |
| **GABRB3** |  |  |  |  |  |  |  |  |  |  |  |  |
|  | rs4906902 | A | 201 | ( | 66.3 | ) | 74 | ( | 67.3 | ) | 0.11 | 0.949 |
|  |  | AG | 95 | ( | 31.4 | ) | 34 | ( | 30.9 | ) |  |  |
|  |  | G | 7 | ( | 2.3 | ) | 2 | ( | 1.8 | ) |  |  |
|  |  | A | 497 | ( | 82.0 | ) | 182 | ( | 82.7 | ) | 0.06 | 0.813 |
|  |  | G | 109 | ( | 18.0 | ) | 38 | ( | 17.3 | ) |  |  |
| **GCH1** |  |  |  |  |  |  |  |  |  |  |  |  |
|  | rs752688 | C | 223 | ( | 72.9 | ) | 72 | ( | 65.5 | ) | 2.51 | 0.285 |
|  |  | CT | 79 | ( | 25.8 | ) | 37 | ( | 33.6 | ) |  |  |
|  |  | TT | 4 | ( | 1.3 | ) | 1 | ( | 0.9 | ) |  |  |
|  |  | C | 525 | ( | 85.8 | ) | 181 | ( | 82.3 | ) | 1.55 | 0.213 |
|  |  | T | 87 | ( | 14.2 | ) | 39 | ( | 17.7 | ) |  |  |
|  | rs3783641 | AA/AT | 73 | ( | 23.9 | ) | 35 | ( | 31.5 | ) | 2.50 | 0.114 |
|  |  | TT | 233 | ( | 76.1 | ) | 76 | ( | 68.5 | ) |  |  |
|  |  | A | 80 | ( | 13.1 | ) | 38 | ( | 17.1 | ) | 2.19 | 0.138 |
|  |  | T | 532 | ( | 86.9 | ) | 184 | ( | 82.9 | ) |  |  |
| **HTR2A** |  |  |  |  |  |  |  |  |  |  |  |  |
|  | rs6311 | CC/CT | 255 | ( | 82.0 | ) | 87 | ( | 78.4 | ) | 0.70 | 0.404 |
|  |  | TT | 56 | ( | 18.0 | ) | 24 | ( | 21.6 | ) |  |  |
|  |  | C | 338 | ( | 54.3 | ) | 109 | ( | 49.1 | ) | 1.80 | 0.179 |
|  |  | T | 284 | ( | 45.7 | ) | 113 | ( | 50.9 | ) |  |  |
|  | rs6313 | AA | 59 | ( | 19.0 | ) | 23 | ( | 20.9 | ) | 0.20 | 0.659 |
|  |  | AG/GG | 252 | ( | 81.0 | ) | 87 | ( | 79.1 | ) |  |  |
|  |  | A | 289 | ( | 46.5 | ) | 112 | ( | 50.9 | ) | 1.29 | 0.256 |
|  |  | G | 333 | ( | 53.5 | ) | 108 | ( | 49.1 | ) |  |  |
| **MAOA** |  |  |  |  |  |  |  |  |  |  |  |  |
|  | rs1137070 | C | 185 | ( | 59.1 | ) | 63 | ( | 56.8 | ) | 2.12 | 0.347 |
|  |  | CT | 94 | ( | 30.0 | ) | 40 | ( | 36.0 | ) |  |  |
|  |  | TT | 34 | ( | 10.9 | ) | 8 | ( | 7.2 | ) |  |  |
|  |  | C | 464 | ( | 74.1 | ) | 166 | ( | 74.8 | ) | 0.04 | 0.848 |
|  |  | T | 162 | ( | 25.9 | ) | 56 | ( | 25.2 | ) |  |  |
| **MTHFR** |  |  |  |  |  |  |  |  |  |  |  |  |
|  | rs1801133 | AA | 57 | ( | 18.4 | ) | 16 | ( | 14.3 | ) | 0.99 | 0.319 |
|  |  | AG/GG | 252 | ( | 81.6 | ) | 96 | ( | 85.7 | ) |  |  |
|  |  | A | 262 | ( | 42.4 | ) | 84 | ( | 37.5 | ) | 1.63 | 0.202 |
|  |  | G | 356 | ( | 57.6 | ) | 140 | ( | 62.5 | ) |  |  |
| **MYT1L** |  |  |  |  |  |  |  |  |  |  |  |  |
|  | rs11127292 | C | 244 | ( | 82.4 | ) | 88 | ( | 82.2 | ) | 0.01 | 0.996 |
|  |  | CT | 49 | ( | 16.6 | ) | 18 | ( | 16.8 | ) |  |  |
|  |  | TT | 3 | ( | 1.0 | ) | 1 | ( | 0.9 | ) |  |  |
|  |  | C | 537 | ( | 90.7 | ) | 194 | ( | 90.7 | ) | 0.00 | 0.981 |
|  |  | T | 55 | ( | 9.3 | ) | 20 | ( | 9.3 | ) |  |  |
| **NOS3** |  |  |  |  |  |  |  |  |  |  |  |  |
|  | rs1799983 | G | 128 | ( | 43.1 | ) | 43 | ( | 39.1 | ) | 1.23 | 0.540 |
|  |  | GT | 125 | ( | 42.1 | ) | 53 | ( | 48.2 | ) |  |  |
|  |  | TT | 44 | ( | 14.8 | ) | 14 | ( | 12.7 | ) |  |  |
|  |  | G | 381 | ( | 64.1 | ) | 139 | ( | 63.2 | ) | 0.06 | 0.800 |
|  |  | T | 213 | ( | 35.9 | ) | 81 | ( | 36.8 | ) |  |  |
| **P2RX7** |  |  |  |  |  |  |  |  |  |  |  |  |
|  | rs2230912 | A | 247 | ( | 79.2 | ) | 87 | ( | 77.7 | ) | 0.92 | 0.631 |
|  |  | AG | 63 | ( | 20.2 | ) | 25 | ( | 22.3 | ) |  |  |
|  |  | G | 2 | ( | 0.6 | ) | 0 | ( | 0.0 | ) |  |  |
|  |  | A | 557 | ( | 89.3 | ) | 199 | ( | 88.8 | ) | 0.03 | 0.861 |
|  |  | G | 67 | ( | 10.7 | ) | 25 | ( | 11.2 | ) |  |  |
| **PCLO** |  |  |  |  |  |  |  |  |  |  |  |  |
|  | rs2522833 | A | 117 | ( | 37.4 | ) | 52 | ( | 46.4 | ) | 3.63 | 0.163 |
|  |  | AC | 149 | ( | 47.6 | ) | 42 | ( | 37.5 | ) |  |  |
|  |  | C | 47 | ( | 15.0 | ) | 18 | ( | 16.1 | ) |  |  |
|  |  | A | 383 | ( | 61.2 | ) | 146 | ( | 65.2 | ) | 1.12 | 0.290 |
|  |  | C | 243 | ( | 38.8 | ) | 78 | ( | 34.8 | ) |  |  |
| **SCN9A** |  |  |  |  |  |  |  |  |  |  |  |  |
|  | rs573542 | C | 3 | ( | 1.0 | ) | 0 | ( | 0.0 | ) | 1.45 | 0.484 |
|  |  | CT | 25 | ( | 8.0 | ) | 11 | ( | 10.0 | ) |  |  |
|  |  | TT | 285 | ( | 91.1 | ) | 99 | ( | 90.0 | ) |  |  |
|  |  | C | 31 | ( | 5.0 | ) | 11 | ( | 5.0 | ) | <0.01 | 0.978 |
|  |  | T | 595 | ( | 95.0 | ) | 209 | ( | 95.0 | ) |  |  |
|  | rs4371369 | A | 56 | ( | 20.9 | ) | 21 | ( | 20.2 | ) | 0.07 | 0.964 |
|  |  | AG | 117 | ( | 43.7 | ) | 47 | ( | 45.2 | ) |  |  |
|  |  | G | 95 | ( | 35.4 | ) | 36 | ( | 34.6 | ) |  |  |
|  |  | A | 229 | ( | 42.7 | ) | 89 | ( | 42.8 | ) | <0.01 | 0.987 |
|  |  | G | 307 | ( | 57.3 | ) | 119 | ( | 57.2 | ) |  |  |
|  | rs4387806 | C | 109 | ( | 43.4 | ) | 31 | ( | 38.8 | ) | 0.74 | 0.690 |
|  |  | CT | 117 | ( | 46.6 | ) | 39 | ( | 48.8 | ) |  |  |
|  |  | TT | 25 | ( | 10.0 | ) | 10 | ( | 12.5 | ) |  |  |
|  |  | C | 335 | ( | 66.7 | ) | 101 | ( | 63.1 | ) | 0.70 | 0.402 |
|  |  | T | 167 | ( | 33.3 | ) | 59 | ( | 36.9 | ) |  |  |
|  | rs4453709 | A | 112 | ( | 37.6 | ) | 40 | ( | 37.4 | ) | 0.38 | 0.827 |
|  |  | AT | 143 | ( | 48.0 | ) | 49 | ( | 45.8 | ) |  |  |
|  |  | TT | 43 | ( | 14.4 | ) | 18 | ( | 16.8 | ) |  |  |
|  |  | A | 367 | ( | 61.6 | ) | 129 | ( | 60.3 | ) | 0.11 | 0.738 |
|  |  | T | 229 | ( | 38.4 | ) | 85 | ( | 39.7 | ) |  |  |
|  | rs4597545 | C | 62 | ( | 19.7 | ) | 22 | ( | 19.8 | ) | 0.05 | 0.976 |
|  |  | CG | 159 | ( | 50.6 | ) | 55 | ( | 49.5 | ) |  |  |
|  |  | G | 93 | ( | 29.6 | ) | 34 | ( | 30.6 | ) |  |  |
|  |  | C | 283 | ( | 45.1 | ) | 99 | ( | 44.6 | ) | 0.01 | 0.904 |
|  |  | G | 345 | ( | 54.9 | ) | 123 | ( | 55.4 | ) |  |  |
|  | rs6746030 | A | 12 | ( | 4.8 | ) | 4 | ( | 4.7 | ) | 0.01 | 0.993 |
|  |  | AG | 100 | ( | 40.0 | ) | 35 | ( | 40.7 | ) |  |  |
|  |  | G | 138 | ( | 55.2 | ) | 47 | ( | 54.7 | ) |  |  |
|  |  | A | 124 | ( | 24.8 | ) | 43 | ( | 25.0 | ) | <0.01 | 0.958 |
|  |  | G | 376 | ( | 75.2 | ) | 129 | ( | 75.0 | ) |  |  |
|  | rs6754031 | G | 33 | ( | 10.6 | ) | 13 | ( | 11.7 | ) | 0.53 | 0.767 |
|  |  | GT | 153 | ( | 49.0 | ) | 50 | ( | 45.0 | ) |  |  |
|  |  | TT | 126 | ( | 40.4 | ) | 48 | ( | 43.2 | ) |  |  |
|  |  | G | 219 | ( | 35.1 | ) | 76 | ( | 34.2 | ) | 0.05 | 0.817 |
|  |  | T | 405 | ( | 64.9 | ) | 146 | ( | 65.8 | ) |  |  |
|  | rs7607967 | A | 51 | ( | 17.0 | ) | 21 | ( | 19.3 | ) | 1.96 | 0.376 |
|  |  | AG | 150 | ( | 50.0 | ) | 46 | ( | 42.2 | ) |  |  |
|  |  | G | 99 | ( | 33.0 | ) | 42 | ( | 38.5 | ) |  |  |
|  |  | A | 252 | ( | 42.0 | ) | 88 | ( | 40.4 | ) | 0.18 | 0.675 |
|  |  | G | 348 | ( | 58.0 | ) | 130 | ( | 59.6 | ) |  |  |
|  | rs12994338 | C | 159 | ( | 53.7 | ) | 53 | ( | 49.5 | ) | 2.06 | 0.358 |
|  |  | CT | 109 | ( | 36.8 | ) | 47 | ( | 43.9 | ) |  |  |
|  |  | TT | 28 | ( | 9.5 | ) | 7 | ( | 6.5 | ) |  |  |
|  |  | C | 427 | ( | 72.1 | ) | 153 | ( | 71.5 | ) | 0.03 | 0.860 |
|  |  | T | 165 | ( | 27.9 | ) | 61 | ( | 28.5 | ) |  |  |
|  | rs13017637 | C | 111 | ( | 38.4 | ) | 37 | ( | 36.3 | ) | 0.34 | 0.843 |
|  |  | CT | 131 | ( | 45.3 | ) | 46 | ( | 45.1 | ) |  |  |
|  |  | TT | 47 | ( | 16.3 | ) | 19 | ( | 18.6 | ) |  |  |
|  |  | C | 353 | ( | 61.1 | ) | 120 | ( | 58.8 | ) | 0.32 | 0.572 |
|  |  | T | 225 | ( | 38.9 | ) | 84 | ( | 41.2 | ) |  |  |
| **SERPINA1** |  |  |  |  |  |  |  |  |  |  |  |  |
|  | rs28929474 | C | 309 | ( | 98.4 | ) | 112 | ( | 100.0 | ) | 1.81 | 0.179 |
|  |  | CT | 5 | ( | 1.6 | ) | 0 | ( | 0.0 | ) |  |  |
|  |  | TT | 0 | ( | 0.0 | ) | 0 | ( | 0.0 | ) |  |  |
|  |  | C | 623 | ( | 99.2 | ) | 224 | ( | 100.0 | ) | 1.79 | 0.180 |
|  |  | T | 5 | ( | 0.8 | ) | 0 | ( | 0.0 | ) |  |  |
| **SLC6A4** |  |  |  |  |  |  |  |  |  |  |  |  |
|  | rs25531 | A | 288 | ( | 92.6 | ) | 102 | ( | 91.1 | ) | 0.27 | 0.604 |
|  |  | AG | 23 | ( | 7.4 | ) | 10 | ( | 8.9 | ) |  |  |
|  |  | G | 0 | ( | 0.0 | ) | 0 | ( | 0.0 | ) |  |  |
|  |  | A | 599 | ( | 96.3 | ) | 214 | ( | 95.5 | ) | 0.26 | 0.611 |
|  |  | G | 23 | ( | 3.7 | ) | 10 | ( | 4.5 | ) |  |  |
|  | rs25532 | C | 241 | ( | 77.2 | ) | 88 | ( | 80.0 | ) | 0.90 | 0.639 |
|  |  | CT | 60 | ( | 19.2 | ) | 20 | ( | 18.2 | ) |  |  |
|  |  | TT | 11 | ( | 3.5 | ) | 2 | ( | 1.8 | ) |  |  |
|  |  | C | 542 | ( | 86.9 | ) | 196 | ( | 89.1 | ) | 0.74 | 0.390 |
|  |  | T | 82 | ( | 13.1 | ) | 24 | ( | 10.9 | ) |  |  |
| **SOD2** |  |  |  |  |  |  |  |  |  |  |  |  |
|  | rs4880 | A | 69 | ( | 22.3 | ) | 24 | ( | 21.6 | ) | 0.03 | 0.984 |
|  |  | AG | 167 | ( | 54.0 | ) | 61 | ( | 55.0 | ) |  |  |
|  |  | G | 73 | ( | 23.6 | ) | 26 | ( | 23.4 | ) |  |  |
|  |  | A | 305 | ( | 49.4 | ) | 109 | ( | 49.1 | ) | 0.00 | 0.948 |
|  |  | G | 313 | ( | 50.6 | ) | 113 | ( | 50.9 | ) |  |  |
| **TAAR1** |  |  |  |  |  |  |  |  |  |  |  |  |
|  | rs8192619 | A | 0 | ( | 0.0 | ) | 0 | ( | 0.0 | ) | 0.91 | 0.342 |
|  |  | AG | 15 | ( | 4.8 | ) | 8 | ( | 7.1 | ) |  |  |
|  |  | G | 299 | ( | 95.2 | ) | 104 | ( | 92.9 | ) |  |  |
|  |  | A | 15 | ( | 2.4 | ) | 8 | ( | 3.6 | ) | 0.88 | 0.348 |
|  |  | G | 613 | ( | 97.6 | ) | 216 | ( | 96.4 | ) |  |  |
| **TACR1** |  |  |  |  |  |  |  |  |  |  |  |  |
|  | rs3771863 | AA/AT | 290 | ( | 97.3 | ) | 103 | ( | 94.5 | ) | 1.91 | 0.167 |
|  |  | TT | 8 | ( | 2.7 | ) | 6 | ( | 5.5 | ) |  |  |
|  |  | A | 497 | ( | 83.4 | ) | 168 | ( | 77.1 | ) | 4.27 | 0.039 |
|  |  | T | 99 | ( | 16.6 | ) | 50 | ( | 22.9 | ) |  |  |
| **TXNRD1** |  |  |  |  |  |  |  |  |  |  |  |  |
|  | rs4964728 | A | 239 | ( | 76.6 | ) | 78 | ( | 70.3 | ) | 2.81 | 0.245 |
|  |  | AG | 64 | ( | 20.5 | ) | 31 | ( | 27.9 | ) |  |  |
|  |  | G | 9 | ( | 2.9 | ) | 2 | ( | 1.8 | ) |  |  |
|  |  | A | 542 | ( | 86.9 | ) | 187 | ( | 84.2 | ) | 0.95 | 0.331 |
|  |  | G | 82 | ( | 13.1 | ) | 35 | ( | 15.8 | ) |  |  |
|  | rs7310505 | A | 43 | ( | 15.6 | ) | 13 | ( | 12.7 | ) | 4.32 | 0.116 |
|  |  | AC | 146 | ( | 52.9 | ) | 66 | ( | 64.7 | ) |  |  |
|  |  | C | 87 | ( | 31.5 | ) | 23 | ( | 22.5 | ) |  |  |
|  |  | A | 232 | ( | 42.0 | ) | 92 | ( | 45.1 | ) | 0.57 | 0.4491 |
|  |  | C | 320 | ( | 58.0 | ) | 112 | ( | 54.9 | ) |  |  |

A, Adenine; C, Cytosine; G, Guanine; T, Thymine.

Acronyms of genes as follows: ADRA1A, adrenoceptor alpha 1A; ADRB2, adrenoceptor beta 2; ADRB3, adrenoceptor beta 3; APOE, apolipoprotein E; BDNF-AS, brain-derived neurotrophic factor antisense RNA; COMT, catechol-O-methyltransferase; CREB1, CAMP responsive element binding protein 1; CRHR1, corticotrophin-releasing hormone receptor 1; DRD3, dopamine receptor D3; DRD4, dopamine receptor D4; FKBP5, FK506 Binding Protein 5; GABRB3, gamma-aminobutyric acid type A receptor beta 3 subunit; GCH1, GTP cyclohydrolase 1; HTR2A, 5-hydroxytryptamine receptor 2A; MAOA, Monoamine oxidase A; MTHFR, methylenetetrahydrofolate reductase; MYT1L, myelin transcription factor 1 like; NOS3, nitric oxide synthase 3; P2RX7, purinergic receptor P2X 7; PCLO, piccolo presynaptic cytomatrix protein; SCN9A, sodium voltage-gated channel alpha subunit 9; SERPINA1, serpin family A member 1; SLC6A4, solute carrier family 6 member 4; SOD2, superoxide dismutase 2; TAAR1, trace amine associated receptor 1; TACR1, tachykinin receptor 1; TXNRD1, thioredoxin reductase 1.
